# Supplementary figures and images for: Functional Analysis of Variance for Association Studies
Source: PLoS One. 2014 Sep 22;9(9):e105074. doi: 10.1371/journal.pone.0105074 (PMC4171465; doi:10.1371/journal.pone.0105074)

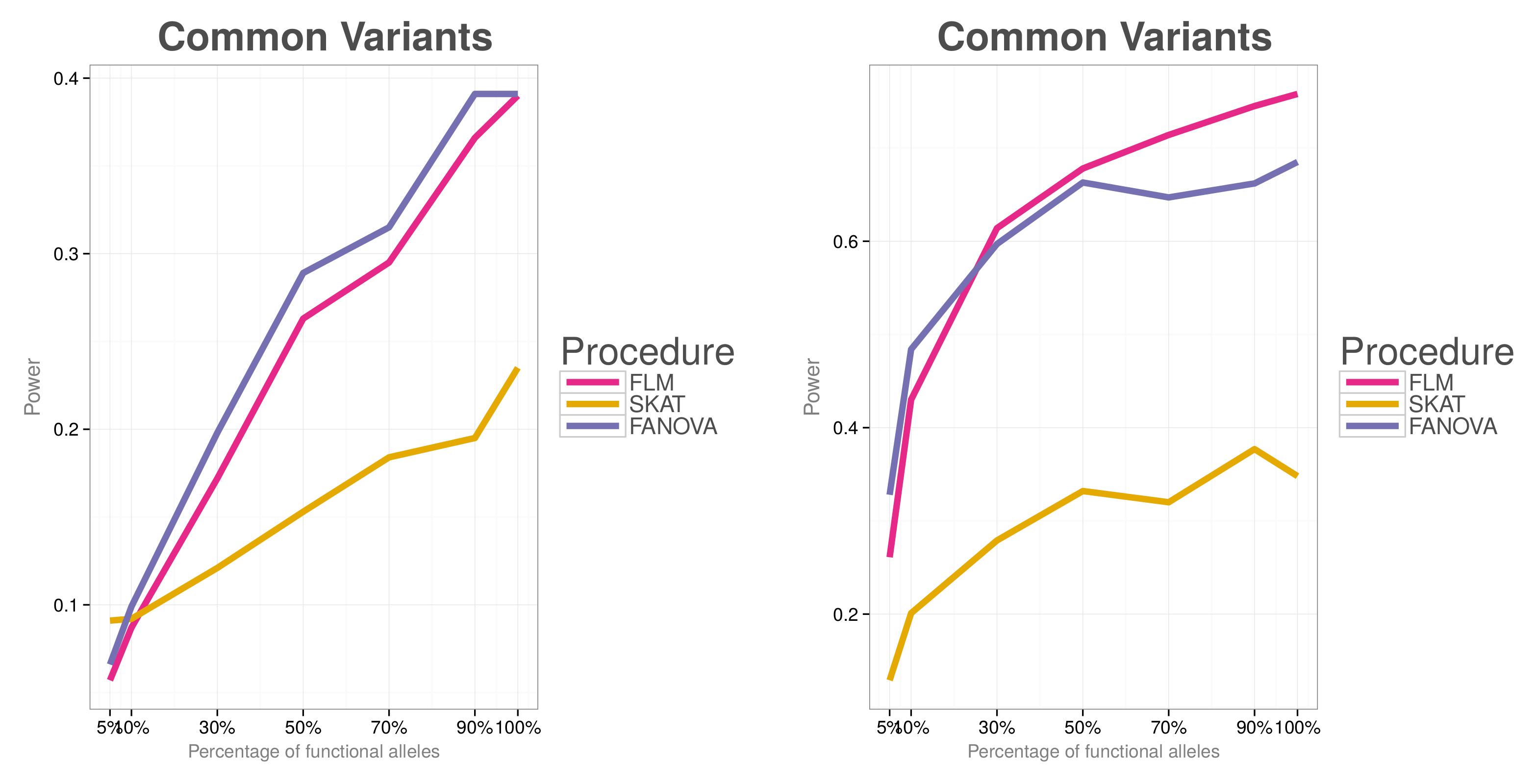

Supplement: Figure S1 — Empirical power of the three methods, only common variants, subjects, first disease model (i.e., ). Left panel: . Right panel: . (TIFF) [file pone.0105074.s001.tiff]

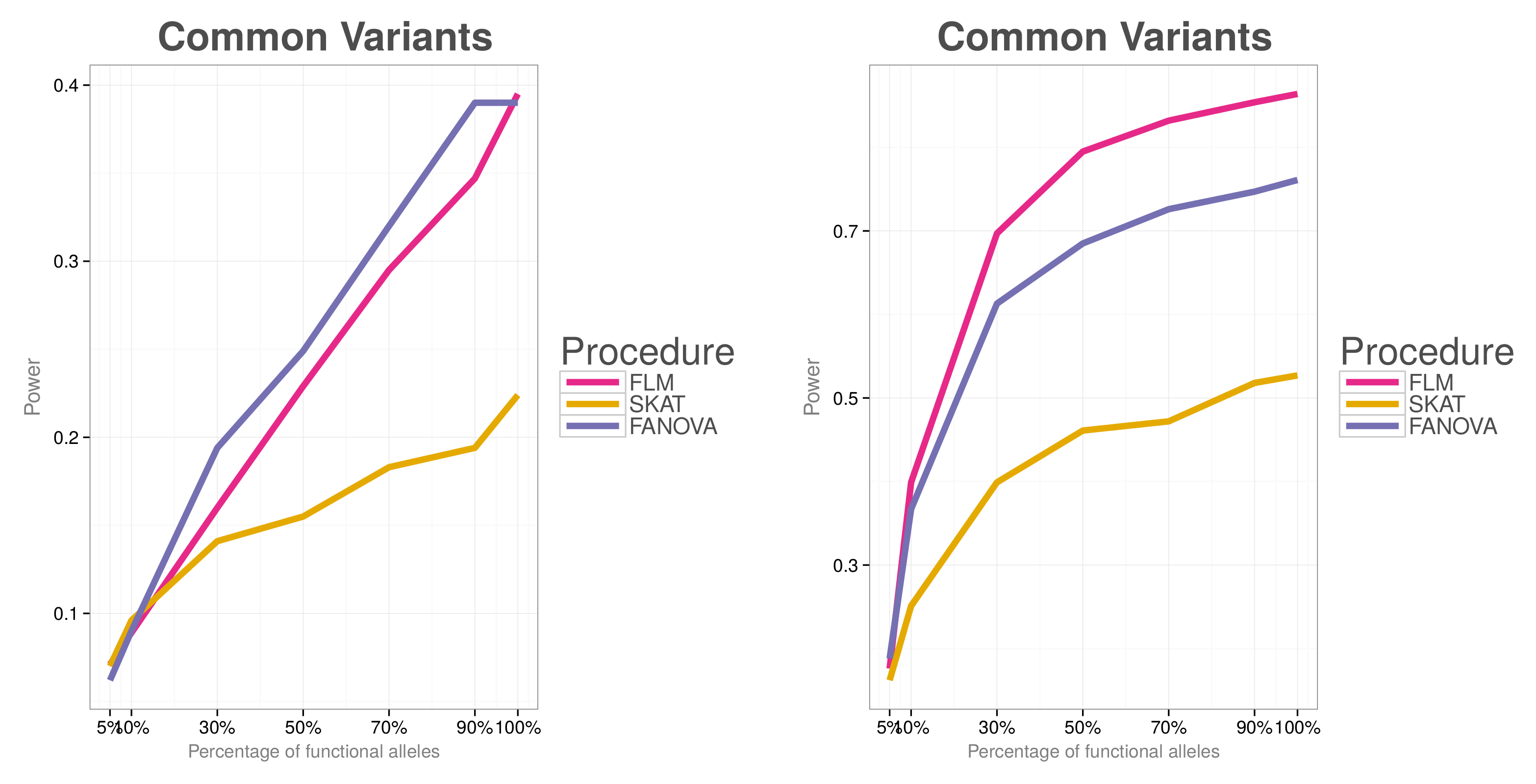

Supplement: Figure S2 — Empirical power of the three methods, only common variants, subjects, second disease model (i.e., ). Left panel: . Right panel: . (TIFF) [file pone.0105074.s002.tiff]

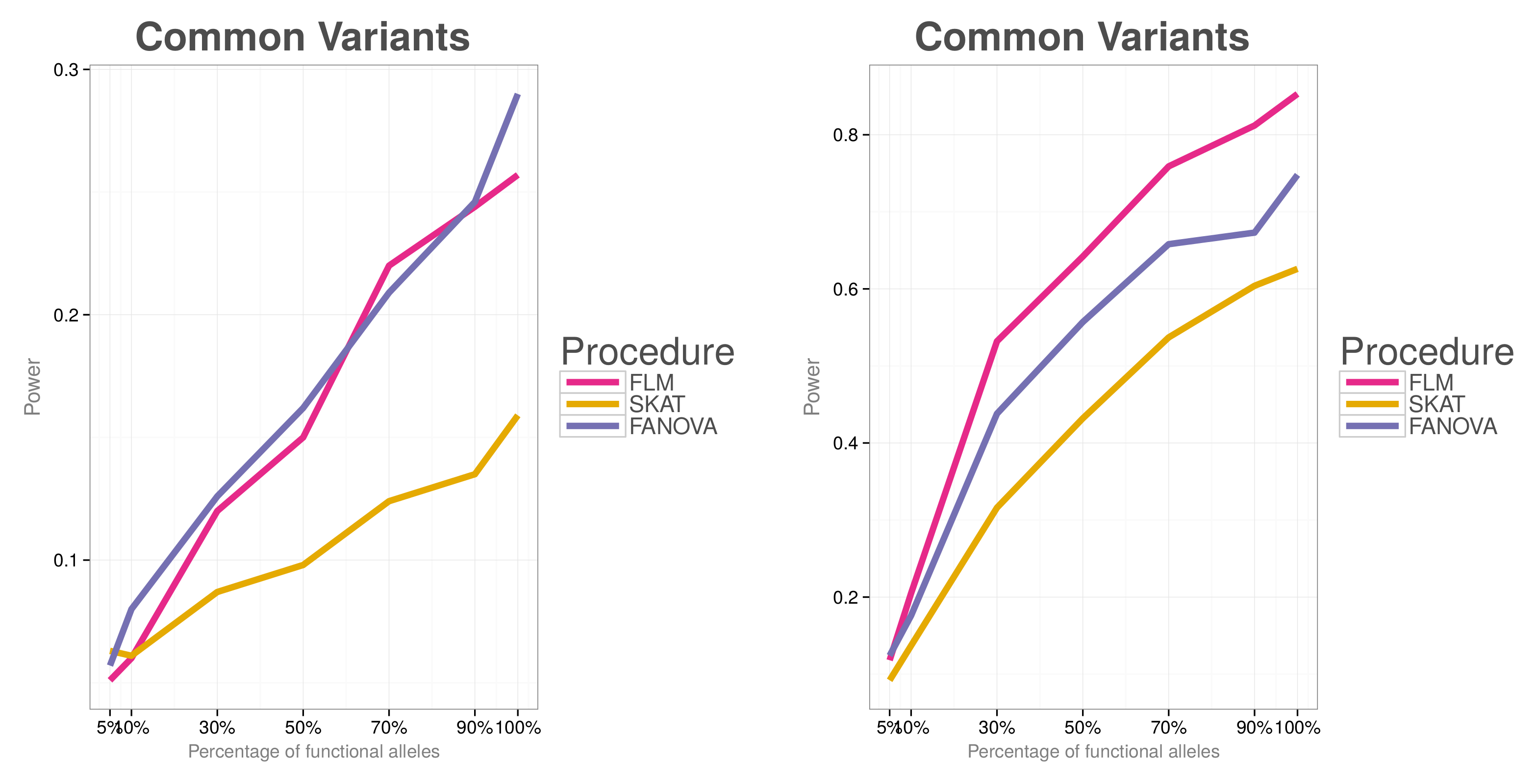

Supplement: Figure S3 — Empirical power of the three methods, only common variants, subjects, first disease model (i.e., ). Left panel: . Right panel: . (TIFF) [file pone.0105074.s003.tiff]

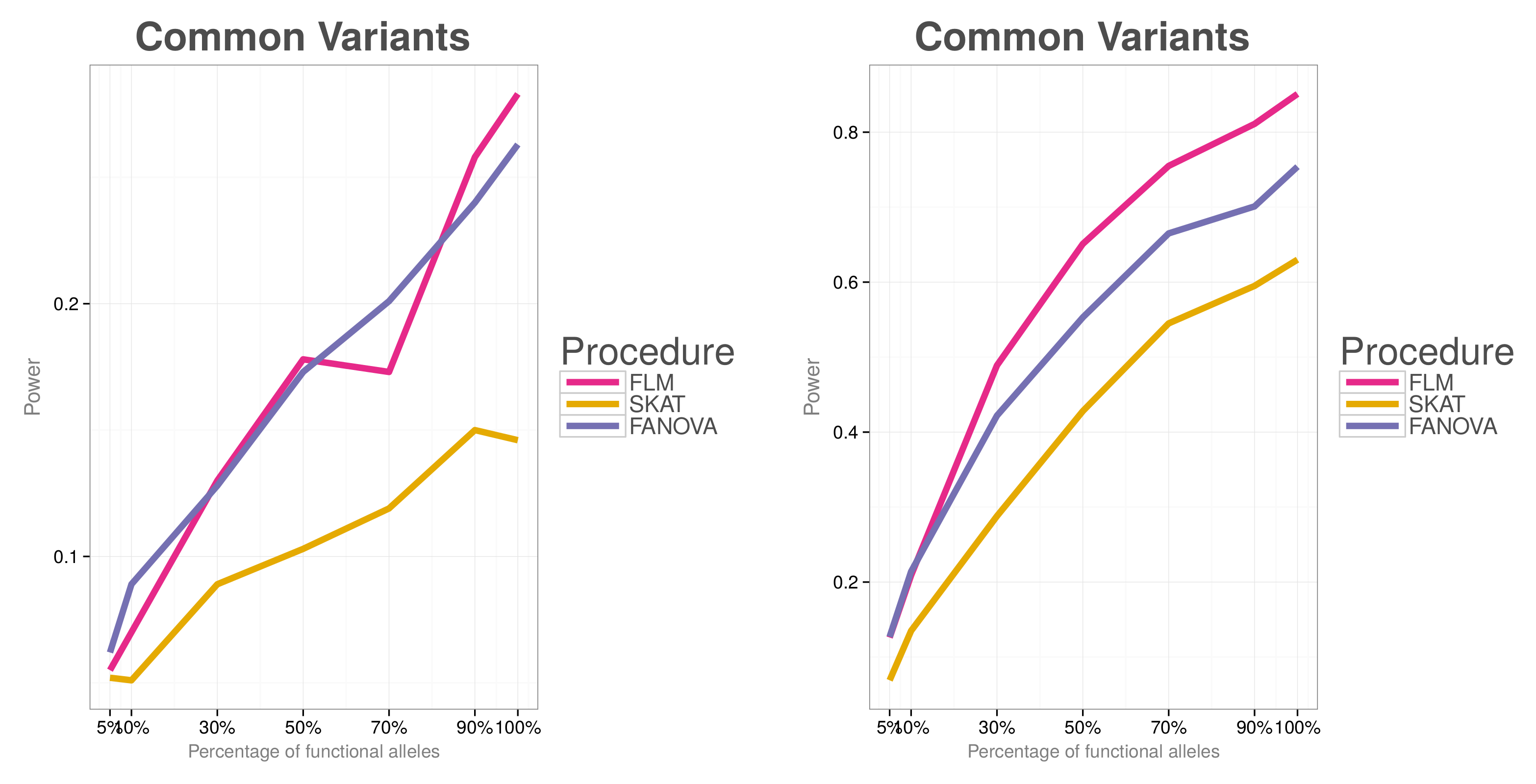

Supplement: Figure S4 — Empirical power of the three methods, only common variants, subjects, second disease model (i.e., ). Left panel: . Right panel: . (TIFF) [file pone.0105074.s004.tiff]

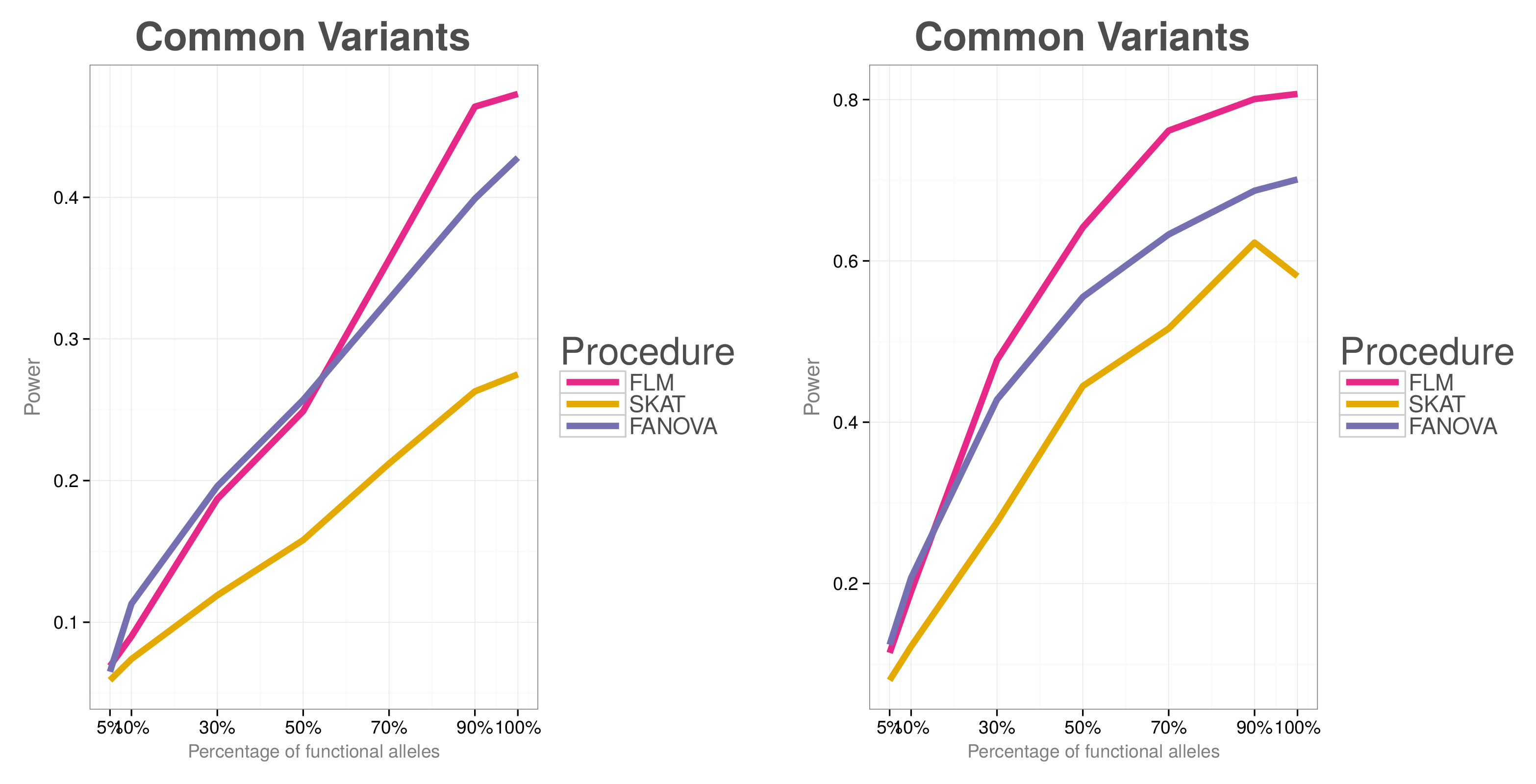

Supplement: Figure S5 — Empirical power of the three methods, only common variants, subjects, first disease model (i.e., ). Left panel: . Right panel: (TIFF) [file pone.0105074.s005.tiff]

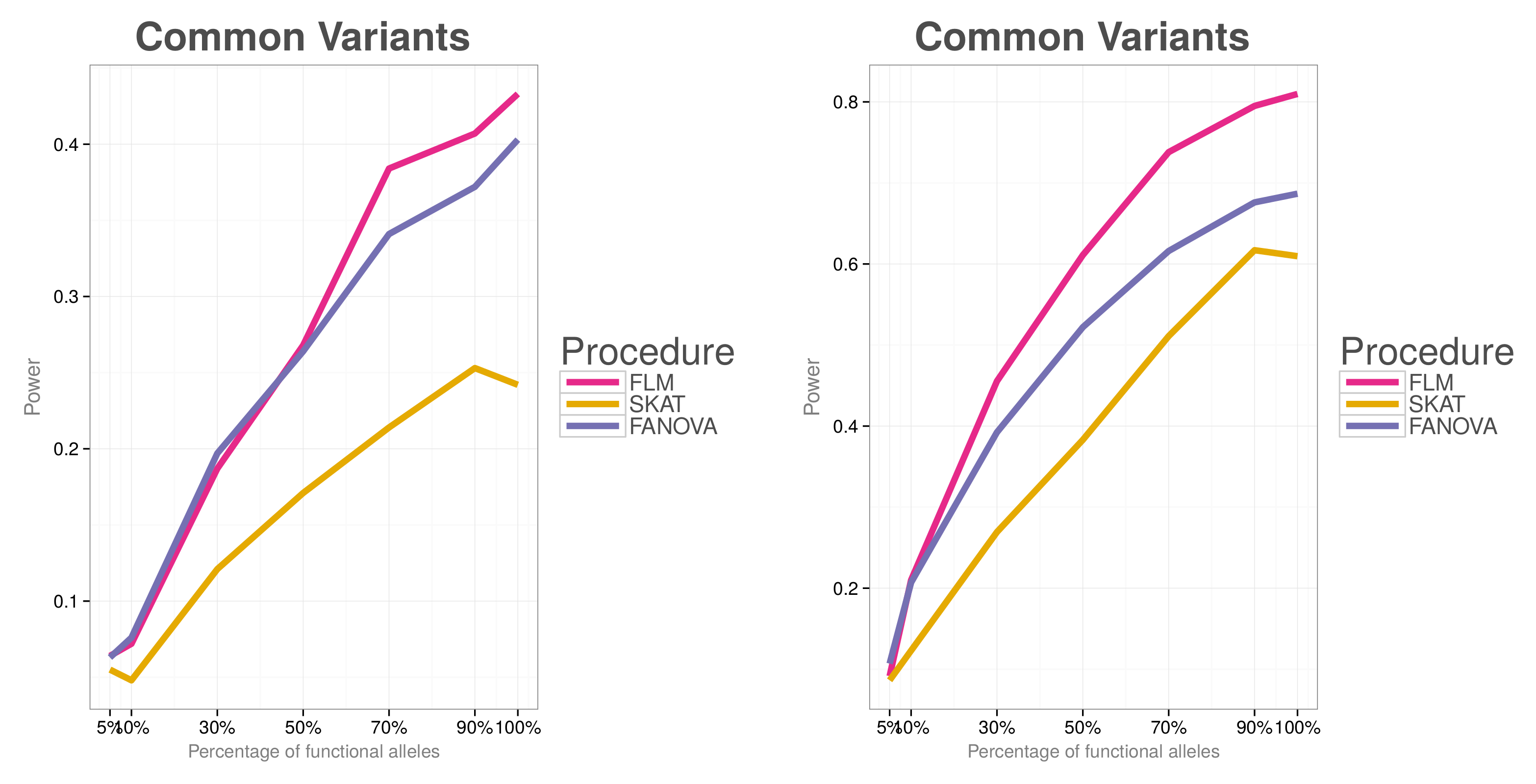

Supplement: Figure S6 — Empirical power of the three methods, only common variants, subjects, second disease model (i.e., ). Left panel: . Right panel: (TIFF) [file pone.0105074.s006.tiff]
